# Effects of 14 frequently used drugs on prostate-specific antigen expression in prostate cancer LNCaP cells

**DOI:** 10.3892/ol.2014.1936

**Published:** 2014-03-05

**Authors:** KAZUHIRO IGUCHI, MAKI HASHIMOTO, MASAFUMI KUBOTA, SHUJI YAMASHITA, MITSUHIRO NAKAMURA, SHIGEYUKI USUI, TADASHI SUGIYAMA, KAZUYUKI HIRANO

**Affiliations:** 1 Laboratories of Drug Metabolism and Pharmacokinetics, Gifu Pharmaceutical University, Gifu 501-1196, Japan; 2 Community Pharmaceutics, Gifu Pharmaceutical University, Gifu 501-1196, Japan; 3 Pharmacy Practice and Social Science, Gifu Pharmaceutical University, Gifu 501-1196, Japan; 4 Drug Informatics, Gifu Pharmaceutical University, Gifu 501-1196, Japan

**Keywords:** prostate-specific antigen, betamethasone, LNCaP

## Abstract

Prostate cancer occurs more frequently among older males and such elderly individuals often have chronic underlying disorders for which various drugs are administered for treatment. The levels of prostate-specific antigen (PSA), a widely used prostate cancer marker, are influenced by a number of drugs, such as non-steroidal anti-inflammatory drugs and statins. In the present study, the drugs prescribed to patients on a repeat prescription collected at the pharmacy of the Gifu Pharmaceutical University (Gifu, Japan) were examined for their effects on the levels of PSA expression in prostate cancer LNCaP cells. Among the 14 drugs investigated, betamethasone, an agonist of the glucocorticoid receptor, was found to increase the levels of PSA mRNA expression in the LNCaP cells. This betamethasone-induced expression was mediated, at least in part, through androgen receptor (AR) transcriptional activation. Dexamethasone, a typical agonist of the glucocorticoid receptor, was also found to stimulate the AR transcriptional activity, however, to a lesser extent than betamethasone. Therefore, it would be interesting to examine in future studies whether the serum PSA levels in prostate cancer patients are influenced by betamethasone.

## Introduction

Prostate cancer is a common disease among elderly men and the prostate-specific antigen (PSA) is a valuable tumor marker for the detection of this cancer. However, improvement of the detection specificity is required as, in addition to prostate cancer, serum PSA levels are also increased in patients with prostate benign hyperplasia and prostatitis (1). Furthermore, it has been recognized that the serum PSA levels are influenced by other drugs, such as statins and non-steroidal anti-inflammatory drugs (2–4). As elderly individuals are frequently prescribed drugs for the treatment of other chronic and underlying illnesses, there is a considerable chance that these drugs may influence the serum PSA levels, subsequently leading to false-positive or false-negative PSA test results (5).

In cases of abnormal growth of prostate cancer cells, the PSA is expressed in prostate epithelial cells and released into the blood by disruption of the basement membrane. Therefore, the potential mechanism underlying medication-induced changes in the blood PSA levels may be that certain drugs induce the release of PSA from the prostate gland or the expression of PSA itself. The present study used prostate cancer LNCaP cells to investigate whether the drugs that are frequently prescribed to patients, and collected at the pharmacy of Gifu Pharmaceutical University (Gifu, Japan), altered the expression level of PSA in prostate cancer LNCaP cells.

## Materials and methods

### Materials

Betamethasone, amlodipine, insulin, lansoprazole, loxoprofen, metformin and warfarin were purchased from Wako Pure Chemical Industries, Ltd. (Osaka, Japan); allopurinol, famotidine, magnesium oxide and D-pantothenic acid were obtained from Nacalai Tesque, Inc. (Kyoto, Japan); aspirin was obtained from Merck Hoei Ltd. (Osaka, Japan); candesartan was purchased from Toronto Research Chemicals Inc. (North York, ON, Canada); and rebamipide was purchased from Tokyo Chemical Industry Co., Ltd. (Tokyo, Japan). All other chemicals used were of analytical grade.

### Investigation of prescription drugs

The prescriptions received at the Gifu Pharmaceutical University pharmacy for one year (between April 1, 2010 and March 31, 2011) were investigated for generic name, dosing period, and patient age and gender. Patients aged between 50 and 75 years at the prescription issue date were the focus of the present study.

### Cell culture

Human prostate carcinoma LNCaP cells were obtained from the American Type Culture Collection (Rockville, MD, USA). The cells were cultured in RPMI-1640 medium containing 10% fetal bovine serum (FBS) and 1% penicillin-streptomycin, under a humidified 5% CO_2_ atmosphere at 37°C.

### Cell viability

Cell viability was evaluated by measuring the fluorescence intensity of cells using the alamarBlue viability assay (Invitrogen Life Technologies, Carlsbad, CA, USA) (6). LNCaP cells were seeded in 96-well plates (Sumilon, Tokyo, Japan) at a density of 8×10^3^ cells/well in RPMI-1640 medium supplemented with 10% FBS. On the following day, cells were treated with various concentrations of each compound and the incubation was continued for three days. AlamarBlue solution was subsequently added to the wells and the plates were incubated for 1 h. Next, the fluorescence intensity of the cells was measured using a POLARstar Galaxy microplate reader (BMG Labtech Ltd., Offenburg, Germany) using excitation and emission wavelengths of 544 and 612 nm, respectively.

### Real-time reverse transcription polymerase chain reaction (qPCR)

qPCR was performed according to previously described protocols with minor modifications (7). Total RNA was extracted using the TRIzol reagent (Invitrogen Life Technologies) and first-strand complementary DNA was synthesized from 1 μg of total RNA using PrimeScript reverse transcriptase (Takara Bio, Inc., Otsu, Japan). Real-time monitoring of the PCR was performed using the Thermal Cycler Dice Real-Time system (Takara Bio, Inc.) with Thunderbird SYBR qPCR mix (Toyobo Corporation, Osaka, Japan). At the end of the reaction, a dissociation curve analysis was performed to examine the specificity of the product. The PCR was performed using the following conditions: 35 Cycles of 15 sec at 95°C and 60 sec at 60°C. The β-actin (*ACTB*) housekeeping gene was used for the normalization of the target mRNA expression. The primers used were as follows: Sense, 5′-GAGGTCCACACACTGAAGTT-3′ and antisense, 5′-CCTCCTGAAGAATCGATTCCT-3′ for PSA (*KLK3*) ; and sense, 5′-CAAGTACTCCGTGTGGATCG-3′ and antisense, 5′-AGTCCGCCTAGAAGCATTTG-3′ for β-actin (*ACTB*).

### Luciferase assay

The luciferase assay was performed as described previously (6). LNCaP cells (1×10^5^ cells/well) were incubated in a 24-well culture plate (Sumilon) for one day and cotransfected with 0.76 μg of the androgen-responsive MMTV-luc firefly luciferase reporter plasmid and 0.04 μg of the *Renilla* luciferase plasmid, phRL-TK, using Lipofectamine 2000 (Invitrogen Life Technologies). Cells were treated for 24 h with various concentrations of betamethasone or dexamethasone. Cell lysates were prepared and the luciferase activities were measured using the Dual-Luciferase Reporter assay system (Promega Corporation, Madison, WI, USA). The firefly luciferase activity was normalized to the activity of *Renilla* luciferase.

### Statistical analysis

Statistical significance was assessed by one-way analysis of variance followed by Dunnett’s test, using PRISM 4 software (Graphpad Software, San Diego, CA, USA). P<0.05 was considered to indicate a statistically significant difference.

## Results

### Effect of the drugs on PSA expression in prostate cancer LNCaP cells

**Figure 1:**
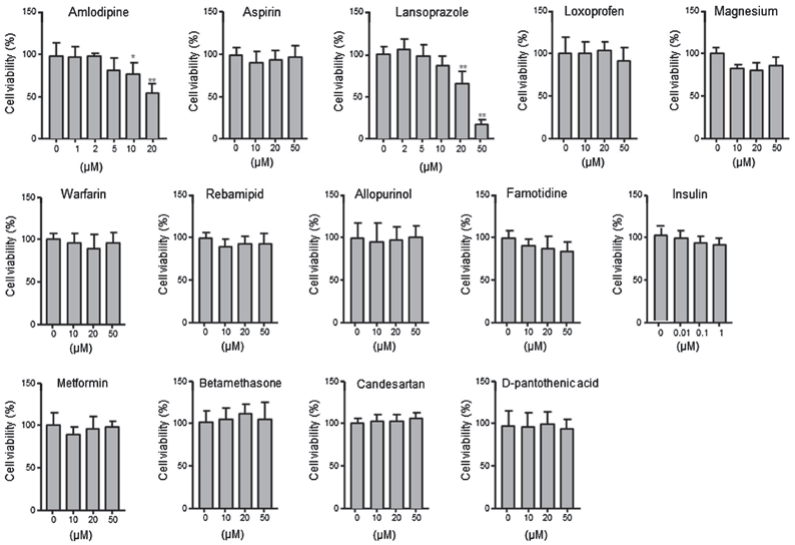
Effects of various drugs on the viability of prostate cancer LNCaP cells. LNCaP cells were treated with various drugs for three days and cell viability was determined by the alamarBlue assay. Data are presented as the mean ± standard deviation of four different incubations. ^*^P<0.05 and ^**^P<0.01 vs. control.

Table I shows the most frequently prescribed drugs for elderly men, including the drugs for the treatment of chronic diseases (such as hypertension and diabetes). Firstly, the effects of the drugs listed in Table 1 on the viability of androgen receptor (AR)-positive prostate cancer LNCaP cells were examined. LNCaP cells were treated with each drug at a concentration of 1–50 μM for 24 h. As shown in Fig. 1, no significant effect was identified on cell viability at concentrations of ≤20 μM, with the exception of amlodipine and lansoprazole. Based on these results, the treatment concentrations were determined to be 2 μM for amlodipine, 10 μM for lansoprazole and 20 μM for the other drugs to examine the effects of the drugs on PSA expression.

**Figure 2:**
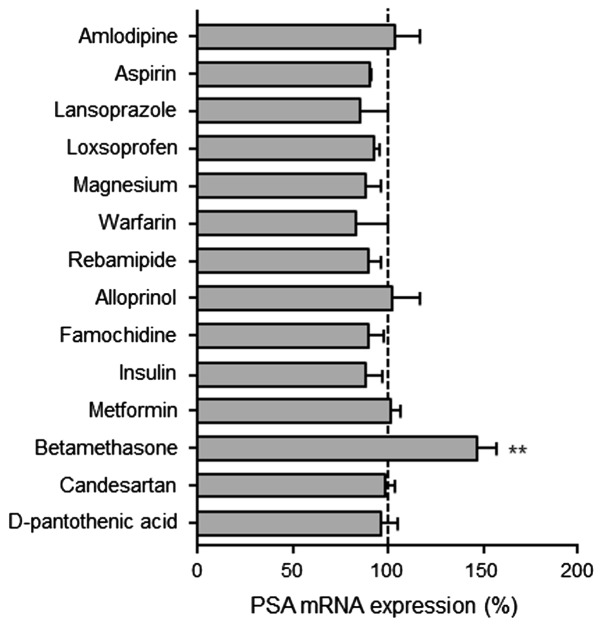
Effect of various drugs on the level of PSA mRNA expression in LNCaP cells. LNCaP cells were treated with 20 μM of each drug for three days with the exception of amlodipine and lansoprazole, which were administered at 2 and 5 μM, respectively. Following incubation, total RNA was isolated and subjected to real-time reverse transcription-polymerase chain reaction analysis. The results were normalized to β-actin (*ACTB)* levels (n=4). ^**^P<0.01 vs. control. The dashed line represents the baseline control (no drug). PSA, prostate-specific antigen.

Fig. 2 shows the PSA mRNA expression in LNCaP cells following treatment with various drugs for three days. The PSA mRNA expression levels increased significantly in the betamethasone-treated LNCaP cells, whereas no significant differences were identified in the levels of PSA mRNA expression among the cells that were treated with the other drugs. Furthermore, long durations of drug treatment (nine or 14 days) showed no significant differences in the PSA expression levels among the drugs that were tested (data not shown).

### Effect of betamethasone on AR transcriptional activity in LNCaP cells

**Figure 3:**
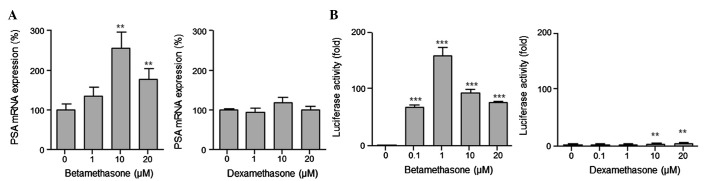
Effects of betamethasone and dexamethasone on the levels of PSA mRNA expression and androgen receptor transcriptional activity in LNCaP cells. LNCaP cells were seeded in phenol red-free RPMI-1640 medium with 2% charcoal stripped fetal bovine serum. (A) Cells were treated with 1–20 μM betamethasone or dexamethasone for three days, after which the total RNA was isolated and subjected to real-time reverse transcription-polymerase chain reaction analysis. The results were normalized to β-actin (*ACTB)* levels (n=4). ^**^P<0.01 vs. control. (B) Following 24 h, the cells were transfected with the MMTV-luc and phRL-TK vectors and treated with 0.1–20 μM betamethasone or dexamethasone for a further 24 h. The cell lysates were prepared and firefly luciferase activity was measured using the Luciferase Reporter assay system and normalized to *Renilla* luciferase activity. ^**^P<0.01 and ^***^P<0.001 vs 0 μM. PSA, prostate-specific antigen.

LNCaP cells have a point-mutated AR (T877A), which broadens the ligand specificity (8,9). Since PSA expression was found to be highly induced by betamethasone (an agonist against the glucocorticoid receptor), which has a steroid structure, this induction was considered to be mediated through transactivation of the mutated AR. As shown in Figs. 2 and 3A (left panel), the PSA induction by betamethasone in steroid-deprived medium was higher than that in normal medium. Furthermore, the AR transcriptional activity measured by the luciferase assay was significantly elevated by betamethasone treatment in the LNCaP cells (Fig. 3B). Notably, dexamethasone, an agonist of the glucocorticoid receptor, did not influence the PSA mRNA expression in the LNCaP cells (Fig. 3A, right panel). Furthermore, although the induction of AR transcriptional activity by dexamethasone was observed, the extent was much lower than that by betamethasone (Fig. 3B, right panel).

## Discussion

The present study examined the drugs most frequently prescribed at the pharmacy of Gifu Pharmaceutical University and found that betamethasone, a frequently used drug among elderly males (rank 12, Table I), increased the PSA mRNA expression in prostate cancer LNCaP cells.

The significant increase of PSA expression by betamethasone is through the transcriptional activation of AR. Betamethasone is a ligand for the glucocorticoid receptor; therefore, the PSA increase is considered to be the result of the agonistic effect of this drug on the mutated AR (T877A) in LNCaP cells. Previously, it has been reported that betamethasone induces AR transcriptional activation to almost the same level as dihydrotestosterone when the T877A AR expression vector is transfected into CV-1 cells, whereas the activation is extremely low with the transfection of wild-type AR (10). In the present study, betamethasone treatment did not activate AR transcription in wild-type AR-transfected PC-3 cells (data not shown). Notably, the induction of AR transcriptional activity by betamethasone was markedly higher than that by the typical glucocorticoid receptor agonist, dexamethasone, in the LNCaP cells that were endogenously expressing T877A AR. A previous study demonstrated no notable differences in the AR transcriptional activity between betamethasone and dexamethasone treatments in T877A AR-transfected CV-1 cells (10). Therefore, the induction of AR transcriptional activity by betamethasone may be involved in a mechanism additional to acting as a ligand to the mutated AR.

It must also be noted that the results from our experimental model using LNCaP cells may provide insight into the effect of drugs on PSA test results. However, the serum PSA levels are not singularly determined by the amount of PSA produced from the cells. In the present study, aspirin did not affect PSA expression in LNCaP cells, although, the serum PSA levels in aspirin users has been identified as lower than those in non-users (4). The lowering of the serum PSA levels by aspirin may be mediated through its anti-inflammatory effect, since chronic inflammation causes the development of prostate cancer and anti-inflammatory aspirin has been postulated to have an inhibitory effect on prostate cancer development.

In the present study, the betamethasone that was prescribed to patients was for external application only. The circulating concentration of an externally applied drug is generally markedly lower than that of a drug that is administered orally. In addition, the PSA increase due to the betamethasone was identified to be particularly significant in the mutated T877A AR, which is consistent with the results of a previous study (10). Occasionally, prostate cancer cells exhibit a mutated AR, particularly in patients who are resistant to androgen ablation therapy (11). Therefore, it appears unlikely that the application of betamethasone to the skin would result in an alternation of serum PSA levels in healthy individuals. However, betamethasone is prescribed to patients with hormone-refractory advanced prostate cancer (12); therefore, in patients that are orally administering betamethasone, a false PSA test result may occur.

In conclusion, the PSA test was identified to be useful for monitoring the disease status in patients with prostate cancer; however, it may be beneficial to examine whether betamethasone influences the PSA test results in clinical cases.

## Figures and Tables

**Table I:** Frequently used drugs from the prescriptions received at the pharmacy of Gifu Pharmaceutical University.

Rank	Generic name	Count, n
1	Amlodipine	107
2	Aspirin	106
3	Lansoprazole	100
4	Loxoprofen	94
5	Magnesium oxide	80
6	Warfarin	74
7	Rebamipide	67
8	Allopurinol	65
9	Famotidine	64
10	Insulin	62
11	Metformin	58
12	Betamethasone	57
13	Candesartan	55
14	D-pantothenic acid	52

Repeat prescription drugs that have been prescribed for >28 days to elderly males (aged 50–75 years).
